# Positive youth development and youth peacebuilding in Colombia

**DOI:** 10.1111/jora.70288

**Published:** 2026-10-13

**Authors:** Vivian Liu, Ellen Kneeskern, Valentina Duarte Valderrama, Claudia Pineda‐Marin, Laura K. Taylor

**Affiliations:** ^1^ University College Dublin Dublin Ireland; ^2^ Fundación Universitaria Konrad Lorenz Bogotá Colombia

**Keywords:** adolescents, civic engagement, Colombia, conflict, peacebuilding, positive youth development, young adults

## Abstract

Positive Youth Development (PYD) is a strengths‐based framework related to young people's well‐being and agency in their environment, which has been recognized as a cornerstone of peaceful societies. Similarly, a growing body of research has documented youth peacebuilding as a way for youth to engage with and foster constructive change interpersonally, structurally, and culturally. Building on recent work, we examine the relationship between PYD and peacebuilding among adolescents and young adults in Colombia, a conflict‐affected country. We analyzed survey data across two time points with Colombian youth (*N* = 806, ages 14–26, 52% male). Four core constructs were used to conceptualize PYD: internal locus of control, prosocial behavior, social trust, and civic accountability. Peacebuilding outcomes were assessed with the Youth Peacebuilding Beliefs Scale, which captures youth beliefs about their roles in promoting peace at different socioecological levels: microsystem (relational change), mesosystem (structural change), and macrosystem (cultural change). First, we explored potential PYD profiles among Colombian youth using latent profile analysis, finding two distinct profiles—youth who were overall high or low in PYD. Second, using path models controlling for age, gender, political orientation, SES, and Time 1 levels for each outcome, we found that greater support for mesosystem peacebuilding was predicted by lower levels of internal locus of control and, at the trend level, lower levels of social trust. Moreover, civic accountability was associated with greater microsystem peacebuilding beliefs. Overall, these findings help understand how diverse beliefs about peacebuilding may be influenced by youth's positive development in Colombia.

## INTRODUCTION

Youth live in a world increasingly marked by conflict, especially those in the Majority World (including Africa, Asia, the Middle East, and Latin America) (UNHCR, 2025). Growing up amidst violent conflict can have a significant negative impact on young people's mental health, socio‐emotional development, and prosocial behavior (Bacchini & Esposito, 2020; Ferrara et al., 2025; Save the Children, 2023; Taylor et al., 2014). Even after formal peace agreements are signed, youth living in “postconflict” societies often face lingering hostility between groups and insecurity (Meger & Sachseder, 2020; Nilsson & González Marín, 2020; Steenkamp, 2005, 2011). In Colombia, for example, nearly 10 years after a historic peace accord ending a 50‐year conflict, there is continued violence, disruption by armed actors, systemic inequality, and limited government support for citizens (Flores & Vargas, 2018; Meger & Sachseder, 2020; Nilsson & González Marín, 2020; The New Humanitarian, 2025). Globally, for the 473 million youth living in conflict‐affected societies (Save the Children, 2023), it is critically important to determine how to mitigate negative effects and promote healthy development.

Positive Youth Development (PYD) is one such avenue, providing a strengths‐based framework for understanding how to foster young people's well‐being, resilience, community engagement, and contributions to peaceful societies (Benson et al., 2011; Lerner, 2004; Lerner et al., 2005, 2011). PYD has been shown to help buffer some of the detrimental effects of conflict and adversity on youth mental health and well‐being (see Lerner et al., 2021 for a review). At the same time, research over the past decade has revealed that youth themselves can play a key role in disrupting conflict and cycles of violence in their communities through active peacebuilding and nonviolent civic engagement (Bähr et al., 2021; Balvin & Christie, 2020; Berents et al., 2024; Del Felice & Wisler, 2007; Taylor, 2020; Taylor et al., 2026). In this paper, we first take a person‐centered approach to explore PYD profiles among Colombian youth using latent profile analysis. Then, we use a variable‐centered approach to examine the links between PYD constructs and youth peacebuilding beliefs, deepening understanding of how youth actively shape a future in which they can thrive.

### Positive youth development

Since its conception in the 1990s, multiple models of PYD have been developed, all of which share a general focus on young people's strengths, developmental plasticity, own internal competencies, and the influence of the external environment on development (Lerner et al., 2011; Qu et al., 2020; Shek et al., 2019). Foregrounding the dynamic interactions between young people and their social contexts, and their strengths rather than deficits, PYD as a framework highlights youths' potential and ability to contribute to their communities, rather than detract from them. These models emphasize constructs typical among thriving youth, such as respect for society, civic engagement and belief in responsibility toward bettering one's community, prosocial behavior, positive connections with peers, families, and communities, confidence that they can overcome obstacles and have a meaningful impact on the world, and belief in their ability to navigate their environment across multiple social levels (e.g., family and school social environments) (Bowers et al., 2010; Geldhof et al., 2013; Heck & Subramaniam, 2009; Lerner et al., 2005, 2011; Qu et al., 2020; Shek et al., 2019).

One of the most well‐known PYD models conceptualizes these constructs as “C's” which are key to positive development and thriving in youth, such as character, connection, confidence, caring, and competence (Lerner et al., 2005). Importantly, this model also includes a 6th “C”, *contribution* to their communities, which comes out of exemplifying the other five C's (Lerner et al., 2005; Shek et al., 2019). Youth who are higher in PYD have a lower risk of internalizing and externalizing behaviors, show higher levels of critical consciousness, and greater engagement with their communities (Geldhof et al., 2015; Lerner et al., 2011). This includes marginalized youth and those living in conflict‐affected environments (see Lerner et al., 2021 for a review).

In our work, we drew on the broader PYD literature (including the five and six C models) to examine how PYD constructs might be related to their peacebuilding beliefs, or contribution to a peaceful society. We examine four PYD constructs in this work, internal locus of control (e.g., Franco & Rodrigues, 2018); prosocial behavior (e.g., Geldhof et al., 2013; Velez‐Gomez et al., 2025), social trust (e.g., Griffith, 2016; Helliwell et al., 2016), and civic accountability (e.g., Flanagan et al., 2007; Law & Atkinson, 2021).

### Youth peacebuilding

Complementing the strengths‐based approach of PYD, another line of research identifies how youth contribute to and improve their societies by disrupting conflict cycles as active peacebuilders (e.g., Taylor et al., 2026; McKeown & Taylor, 2017; Moran et al., 2024; Taylor et al., 2020; Taylor & McKeown, 2017). Peacebuilding is a complex and long‐term process aiming to address the root causes of inequalities that lead to violence, while reducing the risk of lapsing or relapsing conflict in societies (United Nations, 2024). There are two main forms of peacebuilding: episodic, which is aimed at the mitigation and prevention of direct violence against people, and structural, which is aimed at developing sustainable socially just systems and structures in societies which equitably support individuals and their needs (for example, economic needs) (Christie, 2006). In line with this definition, the Developmental Peacebuilding Model (DPM; Taylor, 2020) integrates developmental psychology and peace science to explain the predictors and impact of youth peacebuilding at three different levels: the microsystem, mesosystem, and macrosystem. At the microsystem, peacebuilding consists of interpersonal interactions for relational change, or individual prosocial behaviors, such as helping or sharing (e.g., Bähr et al., 2021; Dirks et al., 2018; McGuire & Rutland, 2020; Padilla‐Walker et al., 2017). At the mesosystem, peacebuilding involves actions for structural change, like collective action to change laws to help disadvantaged groups (e.g., Checkoway & Aldana, 2013; Radke et al., 2020; Taylor & McKeown, 2021). Finally, at the macrosystem, peacebuilding involves actions which strive to create cultural change, like voting behaviors and promotion of social norms aimed at dismantling oppressive systems (Bar‐Tal & Schnell, 2012; Taylor et al., 2024; Townsend et al., 2020).

### Positive youth development and youth peacebuilding

Given the complementary strengths‐based, youth‐focused approach of PYD and youth peacebuilding, recent research has begun to link the two frameworks together (e.g., Johnson et al., 2023; McKeown et al., 2019; McKeown & Taylor, 2017; Yingi, 2023). For example, in Northern Ireland, a postconflict society, McKeown and Taylor (2017) found that among adolescents, higher support for peacebuilding was associated with greater civic engagement, a PYD construct, including volunteering and political participation. In Tanzania, Johnson et al. (2023) examined how PYD components, like sense of community, related to adolescent peacebuilding attitudes and involvement, finding that PYD predicted peace attitudes. Likewise, in a study of youth empowerment programs (through a PYD framework) and peacebuilding in Zimbabwe (Yingi, 2023), it was found that most study participants (18‐ to 35‐year‐olds) believed empowerment programs (especially economic ones) were related to peace in their communities. We contribute to this body of work by examining if PYD relates to peacebuilding in Colombian adolescents and young adults, and exploring the implications for policy frameworks such as the UN's Youth, Peace and Security Agenda (UNSC, 2015, 2018, 2020).

### Colombia as a context

Importantly, while most research on PYD has been conducted in the minority world, key research on PYD in the Majority world is on the rise (e.g., Abdul Kadir & Mohd, 2021; Ataç et al., 2024; Cao et al., 2020; Chen et al., 2017; Desie, 2019; Hull et al., 2021; Marín‐Gutiérrez et al., 2024; Velez‐Gomez et al., 2025). Primarily focused on establishing the validity of PYD in Majority world contexts (e.g., Ataç et al., 2024) and links between PYD and mental health outcomes (e.g., Marín‐Gutiérrez et al., 2024), less work exists on how PYD relates to youth peacebuilding behaviors in their communities. Here, we examine PYD and peacebuilding in Colombia, a Majority world country.

As a conflict‐affected society, Colombian adolescents and young adults are half a generation since the 2016 peace agreement ending the 50‐year civil war, though both regional direct violence (i.e., remaining armed groups) and structural violence (i.e., economic inequality) remain (Gordon et al., 2020; Maher & Thomson, 2018; Meger & Sachseder, 2020). This postconflict environment is shaped by over five decades of internal armed conflict, rooted in deep social inequality, political exclusion, and disputes over land and resources (Jorge et al., 2018; Rettberg, 2020). Given this, Colombia is a particularly relevant context to study intersections between PYD and youth peacebuilding. Despite ongoing political uncertainties, Colombian youth often actively contribute to peace goals, including through youth‐led participatory action research, such as performing arts‐based interventions and the creation of local and national peace murals (O'Sullivan et al., 2023; Taylor et al., 2021). In turn, qualitative research with Colombian youth on their perspectives toward the history of the armed conflict and their position toward the peace accord has highlighted that young people in Colombia often support, engage in, and show emerging orientation and efforts toward communal peacebuilding, intergroup dialogue, forgiveness, and reintegration (Pareja Conto et al., 2024; Velez, 2021, 2022). Examining the underlying constructs (like PYD) behind Colombian youths' peacebuilding beliefs is crucial to understanding how to promote peacebuilding in the future.

### Current study

In this study, we examine two research questions. First, what are the distinct profiles of PYD in Colombian youth (RQ1)? Second, how does each PYD construct relate to youth peacebuilding across different levels (RQ2)?

For RQ1, we first took a person‐centered approach to examining PYD and links with peacebuilding beliefs. Prior research within the PYD framework has predominantly taken variable‐centered approaches (as argued by Buenconsejo et al., 2025), which may obscure distinct patterns of heterogeneity within subgroups of the population if they exist. In contrast, a person‐centered approach (e.g., latent profile analysis) is more consistent with the PYD framework's emphasis on person–context interactions (Lerner et al., 2011) and can reveal additional information on how qualitatively different profiles are associated with unique outcomes (Pivec & Kozina, 2023). Much of the existing person‐centered PYD research has been conducted with WEIRD populations (e.g., the United States and Central Europe; Johnson et al., 2023; Pivec & Kozina, 2023), highlighting an important gap in understanding if or how such profiles may generalize.

We preregistered a four‐profile solution based on the findings of Buenconsejo et al. (2025), who recently identified four distinct profiles of PYD across 21 countries, including a sample in Colombia. We expected the following profiles: (1) low/self‐centered (low across all five PYD constructs), (2) socio‐emotional (lower on internal locus of control but higher on civic accountability, prosocial behavior, and social trust), (3) self‐efficacious (higher on internal locus of control but lower on civic accountability, prosocial behavior, and social trust), and (4) high/balanced (high across all four PYD constructs). Given the relatively exploratory nature of this work, if variability in PYD profiles was lacking, we planned to examine how each PYD construct might relate to youth peacebuilding beliefs, taking a variable‐centered approach. For RQ2, based on the previous literature (e.g., Johnson et al., 2023; McKeown et al., 2019; McKeown & Taylor, 2017; Yingi, 2023), we preregistered the hypotheses of positive associations between each of the PYD constructs and the three levels of the DPM.

## METHOD

### Participants

Participants were 806 Colombian adolescents (*N* = 406, *M* = 15.2, SD = 1.14) and young adults (*N* = 400, *M* = 20.9, SD = 2.10) recruited from Barranquilla (17.6%), Bogota (45.1%), Cali (19.0%), and Medellin (18.3%). The sample was 52% male, 46.5% female, and 1.5% identifying as another gender.

### Procedure

Data were collected from late 2023 to early 2025 as part of a larger longitudinal survey examining adolescent and young adult peacebuilding attitudes and behavior in Colombia. Surveys were administered in Spanish both in person (Time 1) and through phone calls (Time 2), roughly 5–6 months apart by trained research assistants. Participants were recruited from multiple cities across the country using local survey companies. Participation was voluntary, and respondents could withdraw at any time. Surveys took approximately 20–30 min to complete. Ethical approval was obtained from University College Dublin study number (HS‐23‐51‐Taylor), and all participants (and caregivers for minors) provided informed consent/assent prior to participation.

### Measures

#### Predictors: Positive youth development (PYD)

At Time 1, participants were asked to answer questions assessing four different PYD constructs—internal locus of control, prosocial behavior, social trust, and civic accountability—using different scales.

##### Internal locus of control

Internal locus of control was assessed using a seven‐item scale capturing perceived personal control and agency (e.g., “I can do just about anything I really set my mind to”). Responses ranged from 1 (*Strongly disagree*) to 5 (*Strongly agree*) (Lachman & Weaver, 1998), with reverse coded items so that higher values indicate greater personal control. Items were averaged to form a composite internal locus of control score (*α* = 0.68, *ω* = 0.80).

##### Prosocial behavior

Prosocial behavior was measured using a subset of The Child Behavior Scale (Ladd & Profilet, 1996). Participants rated how often they engaged in seven prosocial behaviors (e.g., “I help my peers”). Response options ranged from 1 (*Never*) to 5 (*Always*). Items were averaged to create an aggregate prosocial behavior score (*α* = 0.88, *ω* = 0.91).

##### Social trust

Social trust was measured using two items assessing trust in others in Colombia (Breyer, 2015) (e.g., “In general, most people in Colombia can be trusted”). Responses ranged from 1 (*Strongly disagree*) to 5 (*Strongly agree*), and items were averaged to create a social trust score (*α* = .74).

##### Civic accountability

Civic accountability was assessed using a four‐item civic accountability scale (Flanagan et al., 2007). Items measured youths' sense of civic responsibility and obligation to engage with national and community issues (e.g., “If you love Colombia, you should notice its problems and work to correct them”). Responses ranged from 1 (*Strongly disagree*) to 5 (*Strongly agree*). Items were averaged to create a civic accountability score (*α* = 0.82, *ω* = 0.86).

#### Outcomes: Youth peacebuilding beliefs

Youth peacebuilding beliefs were measured using the Youth Peacebuilding Beliefs Scale (YPBS; Taylor et al., 2026) at Time 1 and Time 2. All items were rated on a 5‐point Likert scale ranging from 1 (*Strongly disagree*) to 5 (*Strongly agree*). The YPBS contains 4 subscales: (1) microsystem peacebuilding (relational change through interpersonal discussions that can “raise awareness” and “change perspectives” about peace), (2) mesosystem peacebuilding (structural change through collective action, such as protests that can “unite” and are an “effective way to build peace”), and (3) macrosystem peacebuilding (capturing cultural change with two subscales of beliefs – voting/politics, such as “voting makes a difference in my country” – and norms, future‐oriented peace narratives, such as “the younger generation has the power to create a more peaceful future”). See Table S1 for the full list of items by subscale.

### Data analytic plan

First, latent profile analysis (LPA) was conducted to identify distinct profiles of PYD based on internal locus of control, prosocial behavior, social trust, and civic accountability at Time 1. Models specifying one through five profiles were estimated using MPlus. Model fit was evaluated using Akaike Information Criterion (AIC), Bayesian Information Criterion (BIC), sample size‐adjusted BIC, entropy, and class size. Model selection prioritized statistical fit, parsimony, and theoretical interpretability.

Second, a path model was used to examine associations between the PYD constructs at Time 1 and youth peacebuilding beliefs at the micro‐, meso‐, and macrosystem using the YPBS at Time 2, controlling for earlier levels of YPBS at Time 1. All PYD constructs and DPM outcomes were included as measured variables to reduce model complexity. Models were estimated using maximum likelihood estimation with robust standard errors (MLR) to account for non‐normality, and maximum likelihood (ML) was used to account for missing data. Model fit was assessed using chi‐square, CFI, TLI, RMSEA, and SRMR, and the LRT to test the structural paths of interest (Chou & Bentler, 1990; Gonzalez & Griffin, 2001).

## RESULTS

Descriptive statistics and bivariate correlations for all study variables are presented in Table S2. Attrition analyses revealed that participants who completed only Time 1 did not differ in terms of demographics (age, gender, political orientation, and SES) or PYD at Time 1 (composite scores for internal locus of control, prosocial behavior, social trust, and civic accountability) compared to those who participated at both timepoints.

### Latent profile analysis of PYD (RQ1)

We explored solutions from one to five profiles and evaluated both fit indices and the interpretability of profile structure to determine the optimal solution (Table 1). AIC, BIC, and sample‐adjusted BIC all decreased with the number of profiles. No profile solution contained a profile of <1.0% of total sample size, meeting the recommended threshold (Lubke & Neale, 2006). Only the two‐ and three‐profile solutions demonstrated an entropy value above the recommended range (.60 to .80; Muthén, 2004; Jung & Wickrama, 2007). Furthermore, visual inspection of the profile means revealed that the three‐profile solution included two profiles with highly similar distributions, suggesting limited differentiation between these profiles (Figure 1).

**TABLE 1:** Model fit indices of latent profile analysis.

Profiles	AIC	BIC	Sample‐adjusted BIC	Entropy	Smallest profile (%)	Largest profile (%)	aLRT	BLRT
1	4626.58	4664.11	4638.71	N/A	100	100	N/A	N/A
2	4448.29	4509.29	4468.00	0.944	5	95	<.001	<.001
3	4389.53	4473.99	4416.83	0.706	4	74	.009	<.001
4	4378.88	4486.79	4413.76	0.575	4	52	.681	<.001
5	4366.64	4498.10	4409.10	0.518	4	37	.031	<.001

*Note*: Based on fit indices and interpretability, a two‐profile solution was deemed most parsimonious.

**FIGURE 1:**
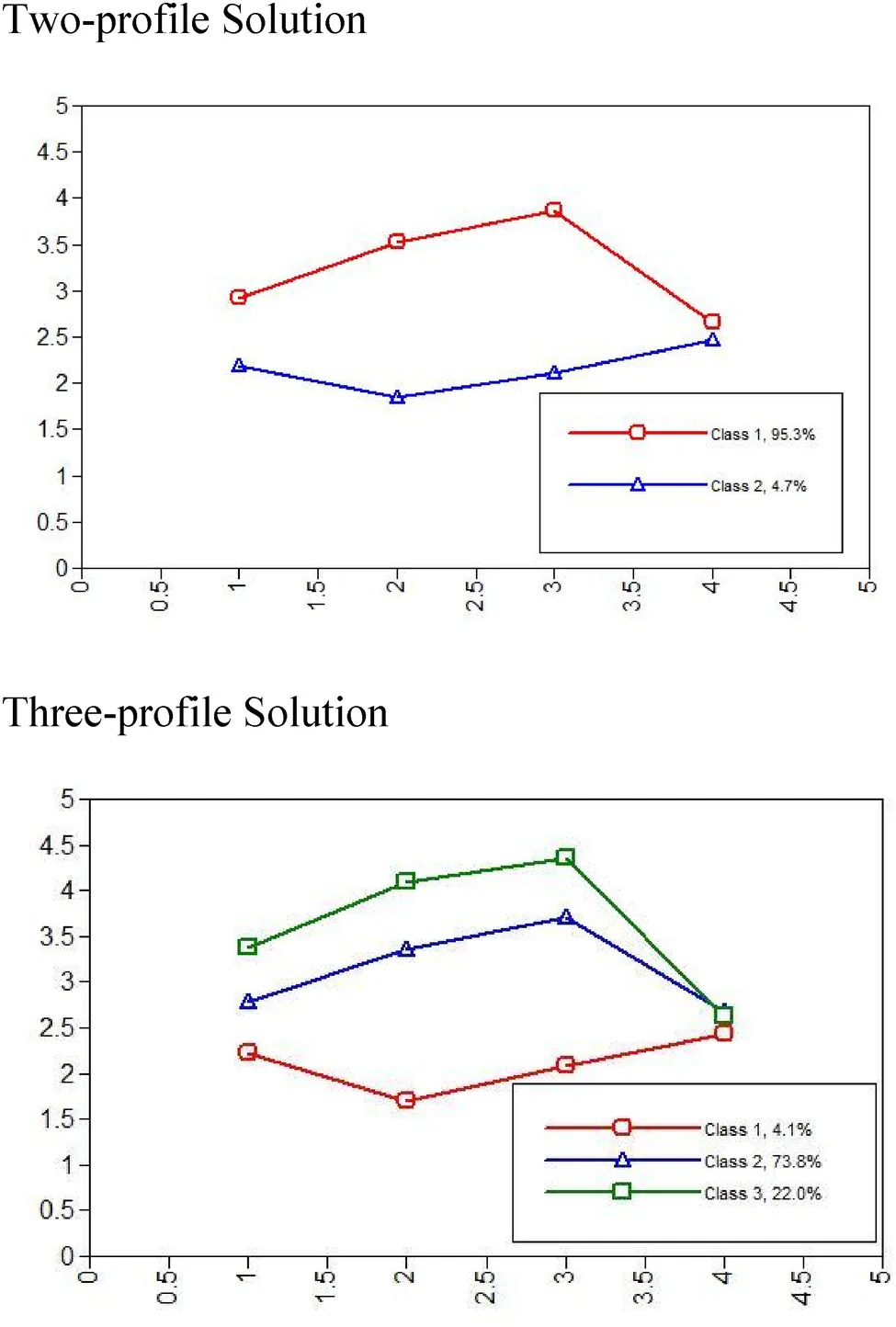
Visualization of the two‐ and three‐profile solutions for the LPA of PYD for Colombian youth (*N* = 806, ages 14–26). The two‐profile solution was chosen, due to similarities in Classes 2 and 3 in the three‐profile solution, and model fit indices (see Table 1).

In contrast, the two‐profile solution demonstrated a clear distinction between one group (High PYD; *n* = 768), scoring higher on internal locus of control, prosocial behavior, and civic accountability, and another group (Low PYD; *n* = 38) scoring lower on these same indicators; scores on social trust were relatively consistent across profiles and clustered around the midpoint of the scale, indicating limited contribution of this indicator to profile differentiation. Taken together, the two‐profile solution was selected as the most parsimonious and theoretically interpretable solution that demonstrated a clear and theoretically meaningful distinction between profiles.

However, although the two‐profile solution provided the most parsimonious and statistically supported fit to the data, this result primarily reflected a general high versus low pattern across the PYD constructs. Such solutions often capture overall level differences rather than qualitatively distinct groups, limiting their theoretical utility. Thus, we proceeded with variable‐centered analyses, using the four PYD constructs as continuous predictors rather than as indicators of profile membership.

### PYD and youth peacebuilding beliefs (RQ2)

Next, we took a variable‐centered approach to testing the relationship between PYD constructs and youth peacebuilding beliefs. As preregistered, we originally tested this relationship using structural equation modeling (SEM) with the youth peacebuilding beliefs subscales specified as latent variables. However, this model did not achieve model fit within acceptable ranges across the required fit metrics (CFI, TLI, RMSEA, and SRMR), so we opted to instead conduct path analysis with observed (measured) variables.

Path models examined associations between each of the constructs at Time 1 (measured; internal locus of control, prosocial behavior, social trust, and civic accountability) and peacebuilding beliefs across four subscales of the YPBS at Time 2 (latent; microsystem, mesosystem, macrosystem voting/politics, and macrosystem norms), controlling for peacebuilding beliefs at Time 1. We report significant and marginally significant paths only in the main text but see Table S3 for coefficients on nonsignificant paths and covariates.

#### Path model

We ran the path model with demographic variables (age, gender, political orientation, and socioeconomic status) as covariates. This model demonstrated good fit to the data, χ^2^(12) = 17.60, *p* = .129; CFI = .981; TLI = .915; RMSEA = .042, 90% CI (.000, .081); SRMR = .015 (Figure 2).

**FIGURE 2:**
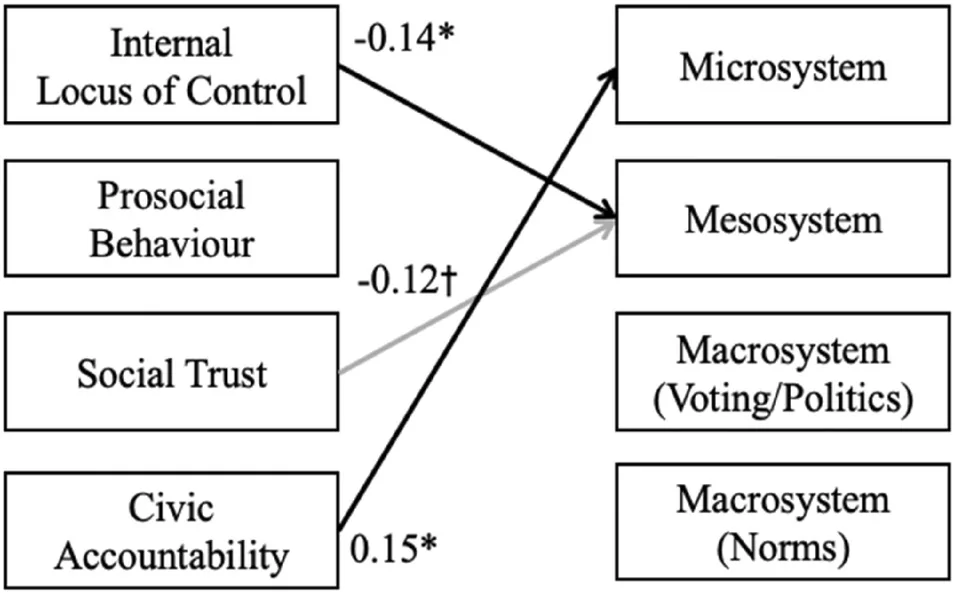
Path model for links between PYD and youth peacebuilding beliefs for Colombian youth (*N* = 806, ages 14–26). PYD constructs were assessed at Time 1. YPBS displayed was assessed at Time 2, controlling for Time 1 levels of each outcome. Demographic covariates (age, gender, political orientation, SES) are included in the model, but not pictured for readability, and allowed to correlate. Black lines indicate significant pathways, gray lines indicate marginally significant pathways. Standardized beta coefficients are reported. *p < .05, ^†^ *p* < .10.

In general, peacebuilding beliefs were higher with age (macrosystem norms, *β* = 0.22, *p* < .001; mesosystem, *β* = 0.13, *p* = .019; microsystem, *β* = 0.19, *p* = .001). Political orientation was negatively associated with macrosystem norms (*β* = −0.13, *p* = .029). No other demographic covariate demonstrated significant relations.

Internal locus of control showed a marginally significant negative association with peacebuilding at the mesosystem level (*β* = −0.14, *p* = .051). Social trust showed a marginally significant negative association with peacebuilding at the mesosystem level (*β* = −0.12, *p* = .096). Civic accountability showed a marginally significant positive association with peacebuilding at the microsystem level (*β* = 0.15, *p* = .051). Prosocial behavior showed no significant associations with any peacebuilding belief outcome (all *p*'s > .10).

Because Wald‐based *p* values can be slightly conservative in this sample size range (Chou & Bentler, 1990), we followed up on all marginally significant paths using 1‐degree‐of‐freedom likelihood ratio tests (LRTs), comparing the full model to a nested model with the parameter of interest fixed to zero (Gonzalez & Griffin, 2001). The LRTs indicated that the paths from internal locus of control to mesosystem, Δχ^2^(1) = 4.13, *p* = .042, and from civic accountability to microsystem, Δχ^2^(1) = 4.32, *p* = .038, were statistically significant, while the path from social trust to mesosystem remained marginally significant, Δχ^2^(1) = 3.65, *p* = .056.

Finally, prior peacebuilding beliefs significantly predicted later beliefs at the macrosystem voting/politics level (*β* = 0.21, *p* = .001), mesosystem level (*β* = 0.21, *p* < .001), and microsystem level (*β* = 0.10, *p* = .047), but not at the macrosystem norms level (*β* = 0.03, *p* = .680).

## DISCUSSION

As frameworks, both PYD and the DPM adopt a strengths‐based approach, focusing on the agentic ways that adolescents and young adults shape their communities for a better future (Lerner et al., 2011; Taylor, 2020). In this study, two distinct findings emerged when we examined links between PYD and youth peacebuilding beliefs in a sample of Colombian youth. First, we found that a two‐profile solution of PYD was the best fit to the data, reflecting general high versus low levels of PYD. Second, we found that internal locus of control, or belief in one's own personal control and agency over their life, was negatively related to mesosystem peacebuilding beliefs, and that civic accountability, or feeling responsibility and obligation to engage with national and community issues, was positively associated with microsystem peacebuilding beliefs. At the same time, there was a marginal negative effect of social trust in others in Colombia on mesosystem peacebuilding beliefs. We explore these findings in turn.

First, addressing RQ1, the best fitting model for PYD in our sample was a two‐profile solution, which ran counter to our hypothesis based on Buenconsejo et al. (2025). In our data, a two‐profile solution suggested that youth were either high or low across prosocial behavior, civic accountability, and internal locus of control, while social trust remained the same, regardless of profile. However, even though the two‐profile solution was the best fit to the data, the vast majority of participants (95%) were in class 1, indicating high PYD. This indicates that despite the conflict‐affected environment they are developing within, on these four PYD constructs, the majority of youth in our sample were well adjusted. However, a small subset (5%) were low on PYD overall. Future work could use measures of PYD to identify at‐risk youth who might need more support to adjust and thrive, particularly in conflict‐affected settings.

Importantly, it is also possible that our measures of PYD may not have captured other types of variability across profiles. Future work could operationalize these constructs differently and examine whether patterns are similar or different by using expanded measures of PYD which might show greater variability, or employing other measures of PYD. For example, in our study we found a marginal association between social trust and mesosystem peacebuilding beliefs. While our social trust measure was in line with other PYD work on social connection (e.g., Griffith, 2016; Helliwell et al., 2016), our measure included only items related to trust in Colombians in general at the country level. Future work could use an extended measure of social trust, teasing it out across family, peer, and local community levels as well (e.g., Lerner et al., 2005), to examine whether this might influence the pattern of results and variability in profiles of PYD.

Second, addressing RQ2, the variable‐centered approach revealed that internal locus of control was significantly related to peacebuilding beliefs at the mesosystem level. The association was negative, suggesting that when youth perceived that they had less personal control and agency, they were subsequently more likely to endorse taking part in protests as a means to express one's beliefs and build peace. In other words, when youth felt like they had less personal control over their own lives and the world around them, they were more likely to believe that structural forms of peacebuilding, such as collective action, were effective means of social change. We underscore here that the outcomes are peacebuilding *beliefs*, and not their own *behaviors*. For example, although youth believe in the efficacy of mesosystem peacebuilding actions, such as protesting as an effective way to build peace, this does not necessarily mean that they are participating in protests themselves. Future work should examine how peacebuilding *behaviors* in addition to *beliefs* might similarly or differentially relate to internal locus of control, to further unravel this effect.

Third, we found that higher levels of civic accountability were positively associated with peacebuilding beliefs at the microsystem level. This finding suggests that when youth believe that active involvement in community issues is their personal responsibility, they are more likely to endorse *interpersonal or relational* change, such as how discussions can raise awareness and change perspectives to promote peace. This finding supports previous research and PYD work; for example, Wray‐Lake and Abrams (2020) found that disadvantaged teens who expressed a personal desire to make a difference in their communities, and those who believed more strongly that you need to work with others to make a difference, were also more likely to engage in civic actions. Our own findings align with and complement this work: youth who endorse a personal responsibility to make a difference in their community also believe that small steps at the interpersonal level can build peace.

Finally, we also found a marginal negative association between social trust and mesosystem peacebuilding beliefs. This finding indicated that, at the trend level, young people in our sample who had lower levels of trust in fellow Colombians were more likely to endorse peacebuilding activities aiming for structural change through collective action. This lack of trust in a conflict‐affected setting like Colombia is not new (e.g., Posada & Wainryb, 2008). In the postaccord period, however, youth who have come out of conflict and may still have lower social trust, also recognize or believe that individuals can work together to change society through social movements.

Our sample of young people was surveyed about 10 years postpeace accord, having spent formative years of their childhood living in a war‐torn country. In this context, youth might have lower feelings of control over their lives. Even so, in our sample, youth with lower levels of internal locus of control still supported agentic behaviors like collective action through protests as a means for structural change, which is in line with both PYD and DPM. Future work should continue to explore these associations and ways to support youth to engage in constructive social movements, particularly among those who may feel a lower sense of personal control or may have lower social trust.

Overall, these findings suggest that to promote peacebuilding in conflict‐affected societies, we should emphasize the impact that youth themselves can have in their communities, which can affect both young people and wider society (Taylor et al., 2021). For example, related participatory action research (e.g., Kustatscher et al., 2022; McGillivray et al., 2025; Uluğ et al., 2023) has been shown to help uplift unheard and marginalized voices for the betterment of their community. Methods like these could be used with Colombian youth to promote civic accountability and protesting to express their opinions, which may have a longer‐term impact on the country's future. However, we caution against over‐interpretation of findings from any one study.

### Limitations and future directions

This work provides initial evidence for links between PYD and youth peacebuilding in Colombia, yet many questions remain. For example, while we included a wide age range (age 14–26), and did see higher levels of peacebuilding beliefs with age, both PYD and peacebuilding have been shown to emerge earlier in development than age 14. Recent PYD work has extended into middle childhood (Moore, 2017; Wiium & Dimitrova, 2019), and the DPM examines peacebuilding even early in childhood (e.g., Bähr et al., 2021; Taylor et al., 2021). Future work could examine the links seen here in a sample of older children or early adolescents, to shed light on links between PYD and peacebuilding at a time in development when significant differentiation in youth's thinking about social issues is occurring (Elenbaas et al., 2020; McGuire et al., 2015).

Further, while we employed a longitudinal design, which controlled for Time 2 outcomes at Time 1, to examine the effect of PYD on later peacebuilding beliefs, our waves were relatively close together. Future work also could replicate this study with additional waves, enabling within‐person estimates of PYD and youth peacebuilding trajectories and their co‐occurrence across these developmental periods.

### Implications for policy and practice

This study contributes to understanding PYD in the Majority world, and in particular Colombia, a conflict‐affected country. The significant links we found from internal locus of control and the marginal link from social trust on mesosystem peacebuilding beliefs are both relevant to the UN's Youth, Peace and Security Agenda (UNSC, 2015, 2018, 2020), specifically SDG16, focused on peace, justice, and strong institutions, and SDG10, focused on reducing inequalities. These goals emphasize that building just governmental institutions and reducing inequalities is critical to promoting peace and justice in society. One such way to do this is to ensure that the right to freedom of expression through collective action is respected. Our findings show that even when young people feel they have a lack of control over their lives, they still might believe that peacebuilding actions, like protests, are effective for constructive social change.

As one key example of the kinds of programs that might continue to be implemented in support of youth agency in peacebuilding in the Colombian context specifically, prior work by Velez examined youth perceptions of a government mandated peace education curriculum focused on young people's role as peacebuilders in their society (Velez, 2021). This work found that adolescents perceived the curriculum's goal of fostering youth empowerment as peacebuilders to be of key importance. Adolescents also indicated that they saw peace education as a way to foster young people's feelings of responsibility to promote peace as citizens of Colombia. This directly relates to our finding that youth who felt a greater sense of civic responsibility also showed greater belief in the importance of interpersonal peacebuilding action. Thus, while more research is needed, education programs in Colombia could continue to focus on youth empowerment and agency as peacebuilders, as they seek to promote greater social harmony.

## CONCLUSION

Our study advances understanding of PYD and its links with peacebuilding in adolescents and young adults in the Majority world. Integrating complementary, strengths‐based frameworks of PYD and the DPM, we found components of each were related across two timepoints. Promoting PYD constructs, such as a sense of civic accountability or personal control, may have implications for peacebuilding at the interpersonal and structural levels, respectively. These types of youth agency and action for constructive social change can both address past wrongs and be future‐oriented through dismantling systemic inequity. Continuing to examine PYD and peacebuilding across multiple developmental periods is an important area of research, with implications for promoting more peaceful societies around the world.

## AUTHOR CONTRIBUTIONS

**Claudia Pineda‐Marin:** Data curation; writing – review and editing; project administration; resources; supervision. **Vivian Liu:** Conceptualization; methodology; investigation; data curation; formal analysis; visualization; writing – original draft; writing – review and editing; project administration; funding acquisition. **Ellen Kneeskern:** Validation; writing – original draft; writing – review and editing. **Valentina Duarte Valderrama:** Investigation; data curation; writing – review and editing; project administration. **Laura K. Taylor:** Conceptualization; methodology; writing – original draft; writing – review and editing; resources; funding acquisition; supervision.

## FUNDING INFORMATION

The research conducted in this publication was funded by Research Ireland under grant number IRCLA/2022/3482 as well as the Society for Research in Child Development Small Grants Program for Early Career Scholars 2024.

## CONFLICT OF INTEREST STATEMENT

The authors declare no conflicts of interest.

## ETHICAL APPROVAL

Ethical approval was obtained from University College Dublin, study number HS‐23‐51, approval date October 19, 2023, and all participants (and caregivers for minors) provided informed consent/assent prior to participation.

## Supporting information


Tables S1–S3.

## Data Availability

For the larger study, we report on how we determined the sample size and exclusion criteria in (https://osf.io/52epw/overview). For this paper, RQ1 measures and hypotheses were preregistered (https://osf.io/hvxk9/overview?view_only=ce61a2eafbe44f3ca5cfce7140bb514a) and RQ2 measures and hypotheses were preregistered (https://osf.io/g8qc2/overview?view_only=5919001318464a0ebc22d362eb084c52). Data and code for both RQ1 and RQ2 are available (https://osf.io/qdx6f/overview?view_only=c74c1d07a2e8463c84870da180d077fb). For RQ1, our sample size was sufficient to detect the correct number of classes based on simulation studies (Tein et al., 2013) and a review article of sample size in LPAs (Spurk et al., 2020). For RQ2, based on the complexity of the proposed model, a simulation study (Wolf et al., 2013) suggests that we have sufficient power to detect the structural paths of interest.

## References

[jora70288-bib-0001] Abdul Kadir, N. B. , & Mohd, R. H. (2021). The 5Cs of positive youth development, purpose in life, hope, and well‐being among emerging adults in Malaysia. Frontiers in Psychology, 12, 641876. 10.3389/fpsyg.2021.641876 PMC8319496 34335359

[jora70288-bib-0003] Ataç, Ö. , Çotuk, M. Y. , & Trejos‐Castillo, E. (2024). Positive youth development approach: A model specific to Türkiye. Applied Research in Quality of Life, 19(3), 1021–1047. 10.1007/s11482-023-10270-0

[jora70288-bib-0004] Bacchini, D. , & Esposito, C. (2020). Growing up in violent contexts: Differential effects of community, family, and school violence on child adjustment. In N. Balvin & D. J. Christie (Eds.), Children and peace. Peace psychology book series. Springer. 10.1007/978-3-030-22176-8_10

[jora70288-bib-0005] Bähr, C. , Dautel, J. B. , Maloku, E. , Razpurker‐Apfeld, I. , Shamoa‐Nir, L. , Humer, J. T. , Misoska, A. T. , & Taylor, L. K. (2021). Helping kids! Cross‐cultural research on children's prosocial behavior in societies transitioning to peace. In Peace psychology book series (pp. 287–303). Springer International Publishing. 10.1007/978-3-030-77688-6_16

[jora70288-bib-0006] Balvin, N. , & Christie, D. J. (2020). Children and peace: From research to action. Springer Nature. 10.1007/978-3-030-22176-8

[jora70288-bib-0007] Bar‐Tal, D. , & Schnell, I. (2012). The impacts of lasting occupation: Lessons from Israeli society. Oxford University Press. 10.1093/acprof:oso/9780199862184.001.0001

[jora70288-bib-0008] Benson, P. , Scales, P. , & Syvertsen, A. (2011). The contribution of the developmental assets framework to positive youth development theory and practice. Advances in Child Development and Behavior, 41, 197–230. 10.1016/b978-0-12-386492-5.00008-7 23259193

[jora70288-bib-0009] Berents, H. , Bolten, C. E. , & McEvoy‐Levy, S. (2024). Introduction. In Manchester university press eBooks. Manchester University Press. 10.7765/9781526176219.00006

[jora70288-bib-0010] Bowers, E. , Li, Y. , Kiely, M. , Brittian, A. , Lerner, J. , & Lerner, R. (2010). The five Cs model of positive youth development: A longitudinal analysis of confirmatory factor structure and measurement invariance. Journal of Youth and Adolescence, 39, 720–735. 10.1007/s10964-010-9530-9 20397040

[jora70288-bib-0011] Breyer, B. (2015). Social Trust Scale (ESS). Zusammenstellung sozialwissenschaftlicher Items und Skalen (ZIS). 10.6102/zis235

[jora70288-bib-0012] Buenconsejo, J. U. , Ferrer‐Wreder, L. , Dimitrova, R. , Pavlova, I. , Bosnar, K. , Bartoluci, S. , Katović, D. , Prot, F. , Sultana, M. S. , Islam, M. S. , Michałek‐Kwiecień, J. , Skrzypińska, K. , Kazmierczak, M. , Lewandowska‐Walter, A. , Borchet, J. , Buzea, C. , Stefenel, D. , Page, D. T. , Luden, M. K. , … Altansukh, S. (2025). Global profiles of positive youth development: A person‐oriented analysis among emerging adults living in 21 countries. Journal of Youth and Adolescence, 54(8), 2094–2119. 10.1007/s10964-025-02174-z 40205131

[jora70288-bib-0013] Cao, J. , Li, X. , Ye, Z. , Xie, M. , & Lin, D. (2020). Parental psychological and behavioral control and positive youth development among young Chinese adolescents: The mediating role of basic psychological needs satisfaction. Current Psychology, 41(7), 4645–4656. 10.1007/s12144-020-00980-1

[jora70288-bib-0014] Checkoway, B. , & Aldana, A. (2013). Four forms of youth civic engagement for diverse democracy. Children and Youth Services Review, 35(11), 1894–1899. 10.1016/j.childyouth.2013.09.005

[jora70288-bib-0015] Chen, B. , Li, X. , & Chen, N. (2017). Positive youth development in China. In Cross‐cultural advancements in positive psychology (pp. 35–48). Springer. 10.1007/978-3-319-68363-8_3

[jora70288-bib-0016] Chou, C. P. , & Bentler, P. M. (1990). Model modification in covariance structure modeling: A comparison among likelihood ratio, Lagrange multiplier, and Wald tests. Multivariate Behavioral Research, 25(1), 115–136. 10.1207/s15327906mbr2501_13 26741976

[jora70288-bib-0017] Christie, D. J. (2006). What is peace psychology the psychology of? Journal of Social Issues, 62, 1–17. 10.1111/j.1540-4560.2006.00436.x

[jora70288-bib-0018] Del Felice, M. C. , & Wisler, A. (2007). The unexplored power and potential of youth as peace‐builder. Peace, Conflict and Development, 11.

[jora70288-bib-0019] Desie, Y. (2019). From surviving to thriving: Characterizing positive youth development among youth in Ethiopia. International Journal of Adolescence and Youth, 25(1), 200–209. 10.1080/02673843.2019.1608272

[jora70288-bib-0020] Dirks, M. A. , Dunfield, K. A. , & Recchia, H. E. (2018). Prosocial behavior with peers: Intentions, outcomes, and interpersonal adjustment. In W. M. Bukowski , B. Laursen , & K. H. Rubin (Eds.), Handbook of peer interactions, relationships, and groups (2nd ed., pp. 243–264). The Guilford Press.

[jora70288-bib-0021] Elenbaas, L. , Rizzo, M. T. , & Killen, M. (2020). A developmental‐science perspective on social inequality. Current Directions in Psychological Science, 29(6), 610–616. 10.1177/0963721420964147 PMC7984411 33758480

[jora70288-bib-0022] Ferrara, P. , Zona, M. , Giardino, I. , Scaltrito, F. , Pettoello‐Mantovani, C. , & Cammisa, I. (2025). The silent wounds of war: Psycho‐physical impacts and international legal implications for children in conflict zones. Global Pediatrics, 14, 100287. 10.1016/j.gpeds.2025.100287

[jora70288-bib-0024] Flanagan, C. A. , Syvertsen, A. K. , & Stout, M. D. (2007). Civic measurement models: Tapping adolescents' civic engagement. CIRCLE Working Paper 55. https://eric.ed.gov/?id=ED497602

[jora70288-bib-0025] Flores, T. E. , & Vargas, J. F. (2018). Colombia: Democracy, violence, and the peacebuilding challenge. Conflict Management and Peace Science, 35(6), 581–586. 10.1177/073889421878778

[jora70288-bib-0026] Franco, G. D. R. , & Rodrigues, M. C. (2018). Self‐efficacy and positive youth development: A narrative review of the literature. Trends in Psychology, 26, 2267–2282. 10.9788/TP2018.4-20En

[jora70288-bib-0027] Geldhof, G. J. , Bowers, E. P. , & Lerner, R. M. (2013). Special section introduction: Thriving in context: Findings from the 4‐H study of positive youth development. Journal of Youth and Adolescence, 42(1), 1–5. 10.1007/s10964-012-9855-7 23139152

[jora70288-bib-0028] Geldhof, G. J. , Bowers, E. P. , Mueller, M. K. , Napolitano, C. M. , Callina, K. S. , Walsh, K. J. , Lerner, J. V. , & Lerner, R. M. (2015). The five CS model of positive youth development. In Advancing responsible adolescent development. Springer. (pp. 161–186). 10.1007/978-3-319-17166-1_9

[jora70288-bib-0029] Gonzalez, R. , & Griffin, D. (2001). Testing parameters in structural equation modeling: Every ‘one’ matters. Psychological Methods, 6(3), 258–269. 10.1037/1082-989X.6.3.258 11570231

[jora70288-bib-0031] Gordon, E. , Henao, S. R. , Duque, A. Z. , & Dolan‐Evans, E. (2020). Power, poverty and peacebuilding: The violence that sustains inequalities and undermines peace in Colombia. Conflict, Security and Development, 20(6), 697–721. 10.1080/14678802.2020.1848119

[jora70288-bib-0032] Griffith, A. N. (2016). Trajectories of trust within the youth program context. Qualitative Psychology, 3(1), 98–119. 10.1037/qup0000049

[jora70288-bib-0033] Heck, K. E. , & Subramaniam, A. (2009). Youth development frameworks. [monograph]. University of California. https://youthrex.com/report/youth‐development‐frameworks/

[jora70288-bib-0034] Helliwell, J. , Huang, H. , & Wang, S. (2016). New evidence on trust and well‐being. 10.3386/w22450

[jora70288-bib-0036] Hull, D. M. , Hinerman, K. M. , Fagan, M. A. , Powell, M. G. , Näslund‐Hadley, E. I. , & Hayes, D. (2021). Positive youth development in Belize: A cluster‐randomised trial of positive action. Educational Psychology, 41(8), 1003–1023. 10.1080/01443410.2021.1922611

[jora70288-bib-0037] Johnson, L. R. , Mudryk, E. , Kibanja, G. M. , Drescher, C. F. , & Malindi, M. (2023). Is positive youth development a pathway for sustaining peace in Tanzania? Predicting peace attitudes among adolescents. Peace and Conflict Journal of Peace Psychology, 29(3), 225–234. 10.1037/pac0000673

[jora70288-bib-0038] Jorge, I. G. M. , Mónica, L. G. V. , Juan, D. L. M. , & Rafael, V. C. (2018). Desigualdad rural y conflicto interno armado en Colombia: un círculo vicioso. Izquierdas, 39, 209–228. 10.4067/S0718-50492018000200209

[jora70288-bib-0039] Jung, T. , & Wickrama, K. A. S. (2007). An introduction to latent class growth analysis and growth mixture modeling. Social and Personality Psychology Compass, 2(1), 302–317. 10.1111/j.1751-9004.2007.00054.x

[jora70288-bib-0041] Kustatscher, M. , Calderón, E. , Tisdall, E. K. M. , Evanko, W. A. , & Gomez Serna, J. M. (2022). Decolonising participatory methods with children and young people in international research collaborations: Reflections from a participatory arts‐based project with Afrocolombian and indigenous young people in Colombia. In Decolonising curriculum knowledge: International perspectives and interdisciplinary approaches (pp. 15–34). Springer International Publishing. 10.1007/978-3-031-13623-8_2

[jora70288-bib-0042] Lachman, M. E. , & Weaver, S. L. (1998). The sense of control as a moderator of social class differences in health and well‐being. Journal of Personality and Social Psychology, 74(3), 763–773. 10.1037//0022-3514.74.3.763 9523418

[jora70288-bib-0043] Ladd, G. W. , & Profilet, S. M. (1996). The child behavior scale: A teacher‐report measure of young children's aggressive, withdrawn, and prosocial behaviors. Developmental Psychology, 32(6), 1008–1024.

[jora70288-bib-0044] Law, A. , & Atkinson, V. (2021). Positive youth development: A bridge to connect civic education and sustainable development. Journal of Youth Development, 16(2–3), 363–378. 10.5195/jyd.2021.1027

[jora70288-bib-0045] Lerner, R. (2004). Liberty: Thriving and civic engagement among America's youth. SAGE Publications, Inc. 10.4135/9781452233581

[jora70288-bib-0046] Lerner, R. , Almerigi, J. , Theokas, C. , & Lerner, J. (2005). Positive youth development: A view of the issues. The Journal of Early Adolescence, 25, 10–16. 10.1177/0272431604273211

[jora70288-bib-0047] Lerner, R. M. , Lerner, J. V. , Lewin‐Bizan, S. , Bowers, E. P. , Boyd, M. J. , Mueller, M. K. , Schmid, K. L. , & Napolitano, C. M. (2011). Positive youth development: Processes, programs, and problematics. Journal of Youth Development, 6(3), 38–62.

[jora70288-bib-0048] Lerner, R. M. , Lerner, J. V. , Murry, V. M. , Smith, E. P. , Bowers, E. P. , Geldhof, G. J. , & Buckingham, M. H. (2021). Positive youth development in 2020: Theory, research, programs, and the promotion of social justice. Journal of Research on Adolescence, 31(4), 1114–1134. 10.1111/jora.12609 34820946

[jora70288-bib-0049] Lubke, G. , & Neale, M. C. (2006). Distinguishing between latent classes and continuous factors: Resolution by maximum likelihood? Multivariate Behavioral Research, 41(4), 499–532. 10.1207/s15327906mbr4104_4 26794916

[jora70288-bib-0050] Maher, D. , & Thomson, A. (2018). A precarious peace? The threat of paramilitary violence to the peace process in Colombia. Third World Quarterly, 39(11), 2142–2172. 10.1080/01436597.2018.1508992

[jora70288-bib-0051] Marín‐Gutiérrez, M. , Caqueo‐Urízar, A. , Castillo‐Francino, J. , & Escobar‐Soler, C. (2024). The 5Cs of positive youth development: Their impact on symptoms of depression, anxiety, stress, and emotional distress in Chilean adolescents. BMC Psychology, 12(1), 372. 10.1186/s40359-024-01863-x PMC11218329 38951933

[jora70288-bib-0054] McGillivray, D. , Guillard, S. , Ross, G. , & McCaughey, P. (2025). Participatory design practice, event (s) and the activation of public space. Journal of Urbanism: International Research on Placemaking and Urban Sustainability, 18(4), 595–612. 10.1080/17549175.2023.2214140

[jora70288-bib-0055] McGuire, L. , & Rutland, A. (2020). Children and adolescents coordinate group and moral concerns within different goal contexts when allocating resources. British Journal of Developmental Psychology, 38(4), 529–542. 10.1111/bjdp.12342 33464607

[jora70288-bib-0056] McGuire, L. , Rutland, A. , & Nesdale, D. (2015). Peer group norms and accountability moderate the effect of school norms on children's intergroup attitudes. Child Development, 86(4), 1290–1297. 10.1111/cdev.12388 26082195

[jora70288-bib-0057] McKeown, S. , Cavdar, D. , & Taylor, L. K. (2019). Youth identity, peace and conflict: Insights from conflict and diverse settings. In Peace psychology book series. Springer International Publishing. (pp. 189–202). 10.1007/978-3-030-22176-8_12

[jora70288-bib-0058] McKeown, S. , & Taylor, L. K. (2017). Intergroup contact and peacebuilding: Promoting youth civic engagement in Northern Ireland. Journal of Social and Political Psychology, 5(2), 415–434. 10.5964/jspp.v5i2.769

[jora70288-bib-0059] Meger, S. , & Sachseder, J. (2020). Militarized peace: Understanding post‐conflict violence in the wake of the peace deal in Colombia. Globalizations, 17(6), 953–973. 10.1080/14747731.2020.1712765

[jora70288-bib-0060] Moore, K. A. (2017). Commentary: Positive youth development goes mainstream. Child Development, 88(4), 1175–1177. 10.1111/cdev.12874 28626930

[jora70288-bib-0061] Moran, D. , Liu, V. , & Taylor, L. (2024). Who, how, and when do children help? A systematic review of children's outgroup prosocial behavior. Political Psychology, 45(S1), 119–155. 10.1111/pops.12948

[jora70288-bib-0062] Muthén, B. (2004). Latent variable analysis: growth mixture modeling and related techniques for longitudinal data. In D. Kaplan (Ed.), Handbook of quantitative methodology for the social sciences (pp. 345–368). SAGE Publications.

[jora70288-bib-0063] Nilsson, M. , & González Marín, L. (2020). Violent peace: Local perceptions of threat and insecurity in post‐conflict Colombia. International Peacekeeping, 27(2), 238–262. 10.1080/13533312.2019.1677159

[jora70288-bib-0064] O'Sullivan, C. , Taylor, L. , Pineda‐Marin, C. , Sandoval‐Escobar, M. , Toro, R. , Barreto, M. L. , Alfonso, D. , & Niño, J. L. (2023). Artesanos de Paz: Promoting everyday peacebuilding among children and youth through a participatory theater‐based intervention in Colombia. Peace and Conflict: Journal of Peace Psychology, 29(3), 213–224. 10.1037/pac0000656

[jora70288-bib-0065] Padilla‐Walker, L. M. , Carlo, G. , & Memmott‐Elison, M. K. (2017). Longitudinal change in adolescents' prosocial behavior toward strangers, friends, and family. Journal of Research on Adolescence, 28(3), 698–710. 10.1111/jora.12362 29144027

[jora70288-bib-0066] Pareja Conto, L. , Velez, G. , Restrepo, A. , Recchia, H. , Posada‐Gilede, R. , & Wainryb, C. (2024). Urban Colombian youths' perspectives on the armed conflict and possibilities for ways forward. Political Psychology, 45(6), 1087–1104. 10.1111/pops.12962

[jora70288-bib-0101] Pivec, T. , & Kozina, A. (2023). Anxiety and COVID‐19 anxiety in positive youth development: A latent profile analysis study. Journal of Youth and Adolescence, 52(11), 2328–2343. 10.1007/s10964-023-01829-z PMC10495271 37500813

[jora70288-bib-0067] Posada, R. , & Wainryb, C. (2008). Moral development in a violent society: Colombian children's judgments in the context of survival and revenge. Child Development, 79(4), 882–898. 10.1111/j.1467-8624.2008.01165.x 18717896

[jora70288-bib-0068] Qu, Y. , Jorgensen, N. A. , & Telzer, E. H. (2020). A call for greater attention to culture in the study of brain and development. Perspectives on Psychological Science, 16(2), 275–293. 10.1177/1745691620931461 PMC9356540 32813984

[jora70288-bib-0069] Radke, H. R. M. , Kutlaca, M. , Siem, B. , Wright, S. C. , & Becker, J. C. (2020). Beyond allyship: Motivations for advantaged group members to engage in action for disadvantaged groups. Personality and Social Psychology Review, 24(4), 291–315. 10.1177/1088868320918698 PMC7645619 32390573

[jora70288-bib-0070] Rettberg, A. (2020). Peace‐making amidst an unfinished social contract: The case of Colombia. Journal of Intervention and Statebuilding, 14(1), 84–100. 10.1080/17502977.2019.1619655

[jora70288-bib-0071] Save the Children International . (2023). Surviving is Just the Beginning: The Impact of Conflict on Children's Mental Health. https://legacy.savethechildren.net/blog/surviving‐just‐beginning‐impact‐conflict‐children‐s‐mental‐health

[jora70288-bib-0072] Shek, D. T. , Dou, D. , Zhu, X. , & Chai, W. (2019). Positive youth development: Current perspectives. Taylor & Francis. https://www‐tandfonline‐com.proxy.uba.uva.nl/doi/full/10.2147/AHMT.S179946 10.2147/AHMT.S179946 PMC6756153 31572041

[jora70288-bib-0073] Spurk, D. , Hirschi, A. , Wang, M. , Valero, D. , & Kauffeld, S. (2020). Latent profile analysis: A review and “how to” guide of its application within vocational behavior research. Journal of Vocational Behavior, 120, 103445. 10.1016/j.jvb.2020.103445

[jora70288-bib-0074] Steenkamp, C. (2005). The legacy of war: Conceptualizing a ‘culture of violence’ to explain violence after peace accords. The Round Table, 94(379), 253–267. https://doi‐org.proxy.uba.uva.nl/10.1080/00358530500082775

[jora70288-bib-0075] Steenkamp, C. (2011). In the shadows of war and peace: Making sense of violence after peace accords. Conflict, Security and Development, 11(3), 357–383. https://doi‐org.proxy.uba.uva.nl/10.1080/14678802.2011.593813

[jora70288-bib-0077] Taylor, L. K. (2020). The developmental peacebuilding model (DPM) of children's prosocial behaviors in settings of intergroup conflict. Child Development Perspectives, 14(3), 127–134. 10.1111/cdep.12377

[jora70288-bib-0076] Taylor, L. , Liu, V. , Corbett, B. , Valderrama, J. V. D. , Eisner, L. , Grütter, J. , Halperin, E. , Hässler, T. , Pineda‐Marin, C. , & Ushomirsky, I. (2026, n.d.). A global youth peacebuilding beliefs scale. 10.1038/s44271-025-00340-4 PMC12886878 41654656

[jora70288-bib-0078] Taylor, L. K. , Marín, C. P. , Cubillos, E. , & Alfonso, D. (2021). YouthLEAD: Measuring the indirect impact of youth peacebuilding through PhotoVoice and community murals in Colombia. In Post‐conflict participatory arts (pp. 153–171). Routledge.

[jora70288-bib-0079] Taylor, L. K. , & McKeown, S. (2017). Youths' peacebuilding potential: Intergroup contact and civic participation amongst a post‐accord generation in Northern Ireland. In Making a difference with psychology (pp. 56–62). Richard Benjamin Trust.

[jora70288-bib-0080] Taylor, L. K. , & McKeown, S. (2021). Adolescent outgroup helping, collective action, and political activism in a setting of protracted conflict. International Journal of Intercultural Relations, 85, 37–46. 10.1016/j.ijintrel.2021.09.001

[jora70288-bib-0081] Taylor, L. K. , Merrilees, C. E. , Goeke‐Morey, M. C. , Shirlow, P. , Cairns, E. , & Cummings, E. M. (2014). Political violence and adolescent out‐group attitudes and prosocial behaviors: Implications for positive inter‐group relations. Social Development, 23(4), 840–859. 10.1111/sode.12074 PMC4594955 26457005

[jora70288-bib-0082] Taylor, L. K. , Merrilees, C. E. , Goeke‐Morey, M. C. , Shirlow, P. , & Cummings, E. M. (2024). Who is building peace? A latent class analysis of youth peacebuilders in a conflict‐affected setting. Political Psychology, 45(5), 853–870. 10.1111/pops.12977

[jora70288-bib-0083] Taylor, L. K. , O'Driscoll, D. , Dautel, J. B. , & McKeown, S. (2020). Empathy to action: Child and adolescent out‐group attitudes and prosocial behaviors in a setting of intergroup conflict. Social Development, 29(2), 461–477. https://doi‐org.proxy.uba.uva.nl/10.1111/sode.12421

[jora70288-bib-0084] Tein, J. Y. , Coxe, S. , & Cham, H. (2013). Statistical power to detect the correct number of classes in latent profile analysis. Structural Equation Modeling: A Multidisciplinary Journal, 20(4), 640–657. 10.1080/10705511.2013.824781 PMC3904803 24489457

[jora70288-bib-0085] The New Humanitarian . (2025). Colombia's surge in armed violence: How did we get here? https://www.thenewhumanitarian.org/analysis/2025/07/31/colombias‐surge‐armed‐violence‐how‐did‐we‐get‐here

[jora70288-bib-0086] Townsend, D. , Taylor, L. K. , Merrilees, C. E. , Furey, A. , Goeke‐Morey, M. C. , Shirlow, P. , & Cummings, E. M. (2020). Youth in Northern Ireland: Linking violence exposure, emotional insecurity, and the political macrosystem. Monographs of the Society for Research in Child Development, 85(4), 7–123. 10.1111/mono.12423 PMC7702086 33184897

[jora70288-bib-0087] Uluğ, Ö. M. , Zoodsma, M. , Sandbakken, E. M. , Figueiredo, A. , Rocha, C. , Sagherian‐Dickey, T. , Acar, Y. G. , Moss, S. M. , Saab, R. , & Woo, Y. T. (2023). How can social psychologists become more participatory in their research? A reflection on working with'communities and participants rather than on'them. Social Psychological Review, 25(1), 9–14. 10.53841/bpsspr.2023.25.1.9

[jora70288-bib-0089] United Nations High Commissioner for Refugees . (2025). Mid‐year trends: Forced displacement in the first half of 2025. United Nations High Commissioner for Refugees.

[jora70288-bib-0090] United Nations Security Council . (2015). Resolution 2250 (2015). https://digitallibrary.un.org/record/814032?ln=en&v=pdf

[jora70288-bib-0091] United Nations Security Council . (2018). Resolution 2419 (2018). https://docs.un.org/en/S/RES/2419(2018)

[jora70288-bib-0092] United Nations Security Council . (2020). Resolution 2535 (2020). https://digitallibrary.un.org/record/3872061?ln=en&v=pdf

[jora70288-bib-0088] United Nations . (2024). Terminology. United Nations Peacekeeping. https://peacekeeping.un.org/en/terminology

[jora70288-bib-0093] Velez, G. (2021). Learning peace: Adolescent Colombians' interpretations of and responses to peace education curriculum. Peace and Conflict: Journal of Peace Psychology, 27(2), 146–159. 10.1037/pac0000519

[jora70288-bib-0094] Velez, G. (2022). Making meaning of peace: A study of adolescents in Bogota, Colombia. Journal of Adolescent Research, 37(3), 439–465. 10.1177/0743558421100691

[jora70288-bib-0095] Velez‐Gomez, P. , Moratto‐Vasquez, N. , Sanchez‐Acosta, D. , & Dodd, S. (2025). Assessing positive youth development in Colombia: Cultural adaptation and validation of a positive youth development scale. International Journal of Applied Positive Psychology, 10(4), 73. 10.1007/s41042-025-00269-3

[jora70288-bib-0096] Wiium, N. , & Dimitrova, R. (2019). Positive youth development across cultures: Introduction to the special issue. Child & Youth Care Forum, 48(2), 147–153. 10.1007/s10566-019-09488-7

[jora70288-bib-0097] Wolf, E. J. , Harrington, K. M. , Clark, S. L. , & Miller, M. W. (2013). Sample size requirements for structural equation models: An evaluation of power, bias, and solution propriety. Educational and Psychological Measurement, 73(6), 913–934. 10.1177/0013164413495 PMC4334479 25705052

[jora70288-bib-0098] Wray‐Lake, L. , & Abrams, L. S. (2020). Pathways to civic engagement among urban youth of color. Monographs of the Society for Research in Child Development, 85(2), 7–154. 10.1111/mono.12415 32394514

[jora70288-bib-0100] Yingi, E. (2023). Youth bulge as a peacebuilding opportunity for Africa: The case of Zimbabwe's youth empowerment programmes. Journal of Asian and African Studies, 60(1), 442–459. 10.1177/00219096231173392

