# Supplementary material for: Positive youth development and youth peacebuilding in Colombia
Source: J Res Adolesc. 2026 Oct 13;36(4):e70288. doi: 10.1111/jora.70288 (PMC13662223; doi:10.1111/jora.70288)
Supplement: Supplementary file 1 [file JORA-36-0-s001.docx]

**Table S1**. Youth Peacebuilding Beliefs Scale (YPBS)

| **Subscale** | **#** | **Item** |
| --- | --- | --- |
| Macrosystem (Beliefs) | 1 | We should care less about group labels and get to know each other as individual people |
|  | 2 | Learning about sectarianism/discrimination is important for peacebuilding |
|  | 3 | Spending time with people from the other background reduces prejudices/negative stereotypes |
|  | 4 | The younger generation has the power to create a more peaceful future |
|  | 5 | Conversations about peace need to focus more on the future and less on the past |
| Macrosystem (Voting/Politics) | 1 | Voting is an important way to express your beliefs |
|  | 2 | Voting makes a difference in my country |
|  | 3 | We need more young people in politics |
| Mesosystem | 1 | Protests are an effective way to build peace |
|  | 2 | Protesting about social issues (e.g., LGBTQ, cost of living) can bring people together |
|  | 3 | Protest art is a way to express your opinion |
| Microsystem | 1 | Discussions can help raise awareness about peacebuilding |
|  | 2 | It [discussions] helps me see a difference perspective |

*Note.* Arranged by subscales.

**Table S2.** Descriptive statistics and bivariate correlations for all study variables

**Table S3.** Structural Equation Model coefficients on non-significant paths and covariates

| YPBS Outcome | *Predictors* | *Estimate* | *SE* | *z* | *p* |
| --- | --- | --- | --- | --- | --- |
| Microsystem | Civic accountability | 0.15 | 0.10 | 1.96 | 0.051† |
|  | Prosocial behaviour | 0.06 | 0.14 | 0.64 | 0.525 |
|  | Social trust | 0.00 | 0.10 | 0.00 | 0.998 |
|  | Internal locus of control | -0.11 | 0.13 | -1.48 | 0.139 |
|  | Age | 0.19 | 0.02 | 3.38 | 0.001** |
|  | Gender | -0.01 | 0.10 | -0.15 | 0.882 |
|  | Political Orien. | -0.09 | 0.05 | -1.55 | 0.121 |
|  | SES | 0.00 | 0.05 | 0.01 | 0.994 |
| Mesosystem | Civic accountability | 0.04 | 0.09 | 0.53 | 0.593 |
|  | Prosocial behaviour | 0.01 | 0.11 | 0.14 | 0.886 |
|  | Social trust | -0.12 | 0.07 | -1.66 | 0.096† |
|  | Internal locus of control | -0.14 | 0.11 | -1.95 | 0.051† |
|  | Age | 0.13 | 0.02 | 2.35 | 0.019* |
|  | Gender | 0.10 | 0.10 | 1.63 | 0.102 |
|  | Political Orien. | -0.01 | 0.04 | -0.20 | 0.842 |
|  | SES | -0.03 | 0.05 | -0.54 | 0.593 |
| Macrosystem | Civic accountability | 0.09 | 0.08 | 1.27 | 0.206 |
| (Voting/Politics) | Prosocial behaviour | 0.11 | 0.12 | 1.34 | 0.180 |
|  | Social trust | -0.13 | 0.09 | -1.42 | 0.157 |
|  | Internal locus of control | -0.12 | 0.12 | -1.63 | 0.103 |
|  | Age | 0.08 | 0.02 | 1.26 | 0.208 |
|  | Gender | 0.00 | 0.11 | 0.05 | 0.963 |
|  | Political Orien. | -0.04 | 0.04 | -0.73 | 0.464 |
|  | SES | 0.03 | 0.05 | 0.45 | 0.655 |
| Macrosystem | Civic accountability | 0.12 | 0.07 | 1.57 | 0.116 |
| (Beliefs) | Prosocial behaviour | -0.04 | 0.12 | -0.36 | 0.722 |
|  | Social trust | -0.05 | 0.07 | -0.55 | 0.580 |
|  | Internal locus of control | -0.05 | 0.09 | -0.63 | 0.526 |
|  | Age | 0.22 | 0.01 | 3.97 | < .001*** |
|  | Gender | -0.03 | 0.08 | -0.50 | 0.616 |
|  | Political Orien. | -0.13 | 0.03 | -2.18 | 0.029* |
|  | SES | -0.07 | 0.04 | -1.16 | 0.247 |

*Note.* † indicates *p* < .10. * indicates *p* < .05. ** indicates *p* < .01. *** indicates *p* < .001.
